# *Houttuynia cordata* Targets the Beginning Stage of Herpes Simplex Virus Infection

**DOI:** 10.1371/journal.pone.0115475

**Published:** 2015-02-02

**Authors:** Pei-Yun Hung, Bing-Ching Ho, Szu-Yuan Lee, Sui-Yuan Chang, Chuan-Liang Kao, Shoei-Sheng Lee, Chun-Nan Lee

**Affiliations:** 1 Department of Clinical Laboratory Sciences and Medical Biotechnology College of Medicine, National Taiwan University, Taipei 100, Taiwan; 2 NTU Center for Genomic Medicine, National Taiwan University College of Medicine, Taipei 100, Taiwan; 3 Department of Laboratory Medicine, National Taiwan University Hospital, Taipei 100, Taiwan; 4 School of Pharmacy, College of Medicine, National Taiwan University, Taipei 100, Taiwan; Cincinnati Childrens Hospital Medical Center, UNITED STATES

## Abstract

Herpes simplex virus (HSV), a common latent virus in humans, causes certain severe diseases. Extensive use of acyclovir (ACV) results in the development of drug-resistant HSV strains, hence, there is an urgent need to develop new drugs to treat HSV infection. Houttuynia cordata (H. cordata), a natural herbal medicine, has been reported to exhibit anti-HSV effects which is partly NF-κB-dependent. However, the molecular mechanisms by which H. cordata inhibits HSV infection are not elucidated thoroughly. Here, we report that H. cordata water extracts (HCWEs) inhibit the infection of HSV-1, HSV-2, and acyclovir-resistant HSV-1 mainly via blocking viral binding and penetration in the beginning of infection. HCWEs also suppress HSV replication. Furthermore, HCWEs attenuate the first-wave of NF-κB activation, which is essential for viral gene expressions. Further analysis of six compounds in HCWEs revealed that quercetin and isoquercitrin inhibit NF-κB activation and additionally, quercetin also has an inhibitory effect on viral entry. These results indicate that HCWEs can inhibit HSV infection through multiple mechanisms and could be a potential lead for development of new drugs for treating HSV.

## Introduction

Herpes simplex virus (HSV) is a double-stranded DNA virus of the *herpesviridae* family. HSV-1 can infect the oral mucosa, eyes, nasal mucosa, oropharynx, and nervous system and may cause severe complications including blindness, atypical pneumonia, aseptic meningitis, encephalitis, and even death [1–3]. HSV-2 is commonly transmitted through the sexual route potentially causing meningitis and encephalitis [4]. Most HSV infections are asymptomatic, but serious systemic symptoms are more often seen in neonates and immune-compromised individuals [5, 6]. The incidence and severity of HSV infections in immunocompromised patients has shown an upward trend in recent years [7–9]. HSV infections are commonly treated with nucleoside analogues such as acyclovir (ACV) and pencyclovir and certain highly bioavailable prodrugs such as valacyclovir and famciclovir [10, 11]. However, these drugs might promote viral mutations leading to drug-resistant HSVs. About 5% of clinical HSV isolates from immunocompromised patients is ACV-resistant [12]. There is an urgent need to develop new drugs against HSV infection.

HSV entry into host cells is divided into three stages [13]. First, viral glycoprotein B (gB) and gC attach to the surface glycosaminoglycans of host cells [14]. Next, gD post-binds to heparin sulfate and other receptors. In the last step, the viral envelope fuses to the plasma membrane of the host cells and penetrates into the cells. Besides blockage of viral entry, another strategy for developing anti-HSV drugs is to regulate the viral gene expression [15]. HSV viral genes are classified into three clusters in the lytic cycle [16]. HSV immediate-early (IE) genes, ICP0, ICP4, ICP22, ICP27, and ICP47, essential for the viral lytic cycle, are first transcribed and responsible for early (E) and late (L) viral gene expressions after viral entry. Certain cellular factors such as OCT1, early growth response 1, and NF-κB are activated after viral penetration and further promote the viral lytic cycle by regulating the expression of viral IE genes [17–19]. NF-κB is recruited onto viral ICP0 promoter to accelerate viral replication in HSV-1 lytic cycle [17]. Thus, modulation of the expression of viral IE genes or cellular factors might shed light on development of therapeutic strategies for HSV infections.

In the past, the development of anti-viral drugs focused mainly on Western medicine. However, it has been noticed recently that some Chinese medicines have anti-viral activities, and over 75% of the antiviral drugs approved from 1981 to 2002 were obtained from natural products [20]. Importantly, some studies have reported that compounds derived from Chinese medicine could block the replication of HSV [21, 22]. N-docosanol, a long chained alcohol, has anti-HSV activity and was approved by the Food and Drug Administration (FDA) as a topical treatment for herpes simplex labialis [21]. Veregen, which in October 2006 was the first botanical medicine approved by the FDA, has been used to treat genital warts caused by human papilloma virus [22]. Thus, Chinese medicine might provide a new roadmap for the development of anti-viral drugs.

*Houttuynia cordata* (*H. cordata*) is a traditional natural Chinese herbal medicine of the Saururaceae family and used for years to relieve lung-related symptoms, such as cough, pneumonia and bronchitis [23, 24]. Many studies revealed the inhibitory effects of *H. cordata* on viral infections, such as HSV, influenza virus, human immunodeficiency virus type 1 (HIV-1), and SARS-CoV [25–29]. Besides anti-viral activities, *H. cordata* also has anti-inflammatory, anti-cancer, and anti-oxidative activities [30, 31]. The major components of *H. cordata* include phenolic compounds, flavones, and alkaloids. The flavonoid components such as quercetin, isoquercitrin, rutin, hyperin, and quercitrin exhibit antioxidant, antimutagenic and free radical scavenging capacity. The phenolic parts including chlorogenic acid have antipyretic and antibiotic activity [32, 33].

It is well known that *H. cordata* water extracts (HCWEs) exhibit anti-HSV activity and the inhibitory activity might affect the NF-κB activation at 6 h.p.i. hereafter which was defined as the second-wave of NF-κB activation in HSV infection [17, 27, 28]. However, the molecular mechanisms involved in anti-HSV activity of HCWEs are not thoroughly explored yet. Whether HCWEs execute the inhibitory activity through targeting virus particles, affecting cellular components or both is still unclear. However, the understanding of mechanism-of-action (MOA) and identification of targets are the most critical requisites in new drug development. In this study, we focused on elucidating MOA of HCWEs in addition to suppressing NF-κB activation and identifying the effective component of HCWEs against HSV infection.

## Materials and Methods

### Virus and cell culture

African green monkey kidney cells (Vero, ATCC CCL-81) and human epithelial carcinoma cells (HEp-2, ATCC CCL-23 and A549, ATCC CCL-185) were maintained in Dulbecco’s modified Eagle’s medium (DMEM), modified Eagle’s medium (MEM) and RPMI-1640 medium, respectively, supplemented with 10% fetal bovine serum (Gibco), 100 units/mL penicillin, and 100 μg/mL streptomycin. All the cell lines were cultured at 37°C with 5% CO_2_. HSV-1F (ATCC VR-733) and HSV-2G (ATCC VR-734) stocks were propagated in Vero cells, aliquoted and stored at -80°C. ACV-resistant HSV-1 was generated in our previous study [34].

### HCWEs preparation

1,000 g dried *H. cordata* was boiled in 2 L water for 3 hours and the supernatant was filtered through a 0.2 um filter and lyophilized into dry powder. The powder was dissolved in double-distilled water to the concentration of 100 mg/ml.

### Plaque reduction assay

1X 10^6^ Vero cells were seeded onto 6-well culture plates for 24 hours and infected with 100 pfu/well virus at 37°C for 1 hour. After incubation, the virus-infected cells were washed twice with phosphate-buffered saline (PBS) and overlaid with 1% methylcellulose containing different concentrations of HCWEs as indicated. After 3 days, the cells were fixed with 3.7% formaldehyde and stained with crystal violet. The plaques were counted.

### Cytotoxicity assay

1X10^4^ cells in 96-well plate were incubated with indicated concentrations of HCWEs at 37°C for 3 days. The cells were washed with PBS, added with 0.1 mg/mL MTT reagent [3-(4,5-dimethylthiazol-2-yl)-2,5-diphenyl tetrazolium bromide; Sigma] and incubated at 37°C for 5 hours. After incubation, MTT reagent was removed and DMSO was added to each well. The absorbance was then determined by ELISA reader (SpectraMAX 340, Molecular Devices) at the wavelength of 550 nm.

### Cell pre-treatment and virus pre-treatment assays

For cell pre-treatment assay, 1X10^6^ Vero cells were pretreated with various concentrations of HCWEs at 37°C for 3 hours. After incubation, HCWEs-pretreated cells were washed with PBS and then infected with 100 pfu HSV. The inhibitory activities were analyzed by plaque assay. For virus pre-treatment assay, 100 pfu HSV was pretreated with various concentrations of HCWEs at room temperature. After 3 hours of incubation, 1X10^6^ Vero cells were infected with HCWEs-pretreated virus and then analyzed by plaque assay.

### Binding and penetration assays

For virus binding assay, 1X10^6^ Vero cells were first cooled to 4°C for 1 hour and exposed to 100 pfu HSV in presence of HCWEs at 4°C for 2 hours. After incubation, the unbound virus and HCWEs were removed with ice-cold PBS and Vero cells were analyzed by plaque assay and Western blot assay. For virus penetration assay, 1X10^6^ Vero cells were cooled to 4°C for 1 hour and then infected with 100 pfu HSV at 4°C to achieve viral binding. The unbound viruses were removed and Vero cells were incubated with HCWEs at 37°C for 10 minutes to allow viral penetration. Vero cells were then washed with neutralization buffer (137 mM NaCl, 2.7 mM KCl, 10 mM Na_2_HPO_4_, 2 mM KH_2_PO_4_, 0.5 M glycine, pH 3.0) for 1 minute to inactivate non-penetrated viruses and analyzed by plaque assay [34, 35].

### Time-of-addition assay

1X10^6^ Vero cells were infected with 1 m.o.i. of HSV at 37°C for 1 hour. After absorption, HCWEs were added to medium at indicated hours-post infection. After 24 hours, total Vero cells were collected and frozen and thawed for three times. Virus titer was determined by plaque assay.

### RNA isolation, reverse transcription, and real-time PCR

RNAs were extracted from virus-infected or mock-infected cells by Trizol reagent (Invitrogen) and treated with DNase I (Promega). 1 μg total RNA was subjected to reverse transcription by SuperScript II First-Strand Synthesis System (Invitrogen) according to the manufacturer’s instructions. The expression level of viral genes was measured by SYBR Green-based real-time RT-PCR. The PCR primer pairs sequences for ICP0 were cited as described [36]. The primer pairs for UL52 and UL13 were UL52F (5’-GACCGACGGGTGCGTTATT-3’)/UL52R (5’-GAAGGAGTCGCCATTTAGCC-3’) and UL13F (5’-GCGACCTGCTGGTCATGTG-3’)/UL13R (5’-TGCGAGCCAATCCTTGAAG-3’).

### Western blot assay

Cells were harvested in RIPA lysis buffer (50 mM Tris-HCl [pH 7.4], 150 mM NaCl, 1 mM EDTA, 1% Triton X-100, 0.1% SDS, 1% sodium deoxycholate, 1 mM PMSF, and protease inhibitor cocktail) and the protein concentration was measured by the BCA protein assay (BioRad). Proteins were resolved by 12.5% sodium dodecyl sulfate polyacrylamide gel electrophoresis, transferred onto PVDF membrane, blocked with 5% skimmed milk in Tris-buffered saline (TBS) (20 mM Tris-HCl [pH 7.5], 150 mM NaCl, and 0.5% Tween-20) and reacted with primary antibodies for β-actin (1:5000; Sigma), VP16 (1:200; Santa Cruz), ICP4 (1:200; Santa Cruz) and NF-κB (1:200; Santa Cruz). β-actin acts as an internal control.

### Plasmid construction

HSV-1 ICP0 promoter region was amplified from HSV-1 viral DNA using the following primer set (ICP0pF: 5’-CTCGAGTTATGCTAATTGCTTTTTTGG-3’ and ICP0pR: 5’-AAGCTTTCGTATGCGGCTGGAGGG-3’). The primer set (mutF: 5’-GGAAGGCAAAGGAAAAAGGGGCAC-3’ and mutR: 5’-GTGCCCCTTTTTCCTTTGCCTTCC-3’) was used to perform PCR-based mutagenesis by which ten mutant nucleotides were introduced in the NF-κB binding site within viral ICP0 promoter. Both PCR fragments were cloned into pGL3.0 basic vector (Promega).

### Luciferase assay

All transfections were carried out in triplicate in 96-well plates. 2X10^3^ cells were seeded 24 hours prior to transfection. The luciferase reporter constructs along with the control plasmid (pRL-TK Vector; Promega) were co-transfected into cells. After 24 hours, transfectants were infected with HSV at 1 m.o.i.. The luminescent signals were measured at 48 h.p.i. by Victor^3^ multilabel counter (PerkinElmer) according to the manufacturer’s instructions. The activity of *Renilla* luciferase was used as an internal control to normalize transfection efficiency.

### Compounds preparation

The pure compounds, quercetin, isoquercitrin, quercitrin, hyperin, rutin and acyclovir (ACV), were purchased from Sigma-Aldrich, dissolved in dimethyl sulfoxide (DMSO) or absolute ethanol and diluted with sterile deionized distilled water before use.

## Results

### HCWEs inhibit HSV replications

Previous studies have indicated HCWEs exhibit a promising anti-virus activity against HSV-1 and HSV-2 in Vero and BCC-1/KMC cells [27, 28]. These studies shed light on a new direction for development of anti-HSV drugs, however, the MOA is not well understood. Before we performed this study we first measured the anti-HSV activity of HCWEs because the activity may be different from previous reports due to the different sources of *H. cordata* and different extraction procedures [27, 28]. The cytotoxicity of HCWEs was measured by MTT assays. Vero cells were treated with serial dilutions of HCWEs at 37°C for 72 hours. As shown in S1 Table, HCWEs showed low cytotoxicity on Vero cells (CC_50_ was greater than 100 mg/ml). Next, the anti-HSV activity of HCWEs was determined by the plaque reduction assay. The results indicated that EC_50_ of HCWEs against HSV-1, Acyclovir-resistant HSV-1 (HSV-AR) and HSV-2 were 0.692 mg/ml, 1.11 mg/ml and 0.3 mg/ml, respectively (S1 Table). Intriguingly, the EC_50_ of HCWEs against HSV-AR was similar to that against wild-type HSV virus, and this fact implied that the antiviral mechanisms of HCWEs and ACV might be different. The selective index (SI) scores of HCWEs to HSV-1, HSV-AR, and HSV-2 were 144.51, 90.09, and 333.33 respectively. The inhibitory effects of ACV on HSV were also analyzed, and the EC_50_ of ACV against HSV-1, HSV-AR, and HSV-2 were 0.74 μg/ml, 14.02 μg/ml, and 0.82 μg/ml respectively (S1 Table). We demonstrated self-prepared HCWEs used here could inhibit HSV infection as previous reports [27, 28].

### HCWEs inhibit HSV infections by targeting virus particles directly

**Figure 1:**
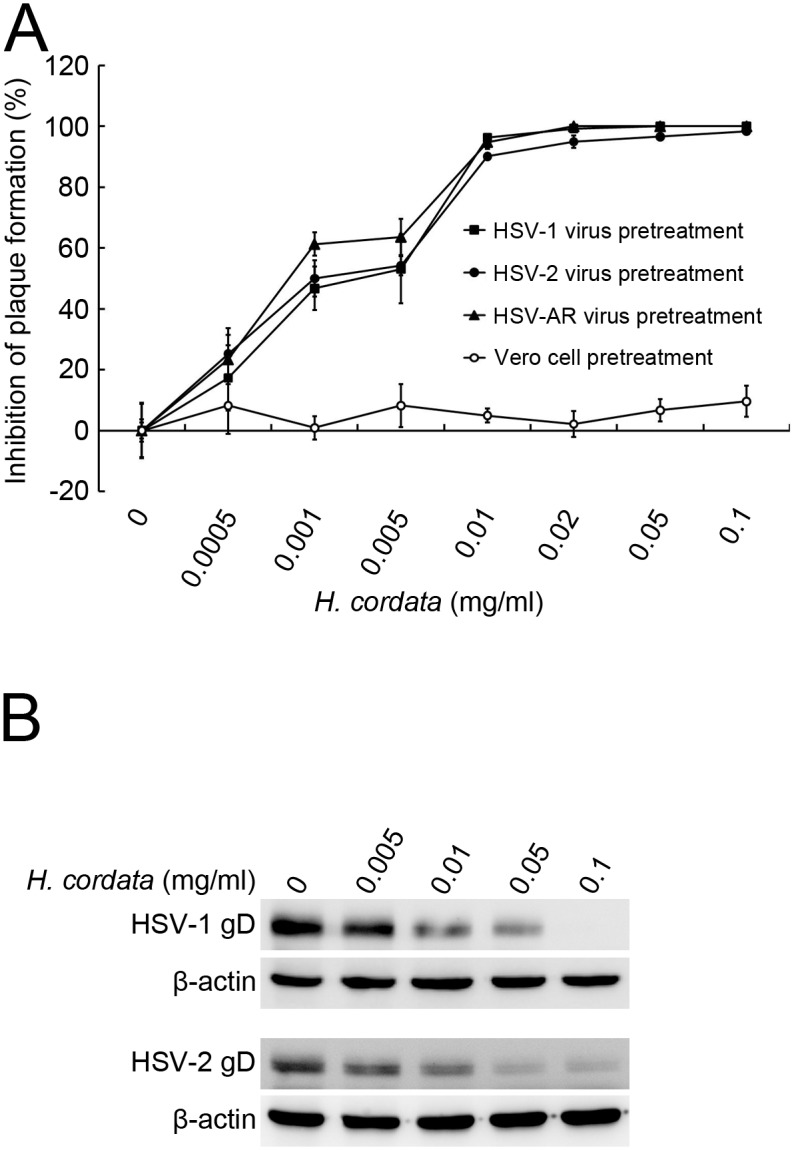
HCWEs block virus binding to host cells via targeting viral particles directly. **(A)** HCWEs inhibited plaque formation in pretreated virus but not in pretreated cells. In the virus pretreatment assay, 1X10^6^ Vero cells were seeded onto 6-well plates and infected with 100 pfu HSV-1 (filled square), HSV-2 (filled circle), and HSV-AR (filled triangle) pretreated with various concentrations of HCWEs as indicated and analyzed by plaque assays. In the cell pretreatment assay, 1X10^6^ Vero cells were pretreated with indicated concentrations of HCWEs followed by 100 pfu HSV-1 infection and then analyzed by plaque assays (open circle). **(B)** HCWEs blocked HSV-1 and HSV-2 binding to Vero cells. 1X10^6^ Vero cells were incubated with HSV at 1 m.o.i. at 4°C in the presence or absence of HCWEs, and the unbound virus was washed with ice-cold PBS. The gD of cell-bound HSV-1 was detected by Western blot assays.

To determine which stage HCWEs hit and identify which viral proteins or host factors mediated the anti-virus activity, we first explored whether HCWEs lead host cells to initiate anti-viral mechanisms or interfere virus particles directly to block further viral binding and penetration, Vero cells and HSV were pretreated respectively with HCWEs and the plaque reduction assay was performed to determine which stage HCWEs act on HSV infection. The pretreated Vero cells showed no obviously inhibitory effect on HSV-1 infection at a concentration of 10 mg/ml (S1 Fig.), which is 14 times higher than its EC_50_ (0.692 mg/ml). However, in the cases of HSV-1, HSV-2, and even HSV-AR pretreated with 0.01 mg/ml HCWEs, the plaque formation was largely inhibited (Fig. 1A). These results implied that the anti-HSV effects of HCWEs might target virus particles directly and inhibit further stages of HSV infection. To examine this hypothesis, HCWEs-pretreated HSV-1 and HSV-2 were incubated with Vero cells at 4°C to allow virus to bind onto cells but not penetrate the cells. The unbound viruses were washed out with PBS, and the cell-bound viruses were analyzed by Western blot analysis. HCWEs obviously decreased the cell binding ability of HSV-1 and HSV-2 in a dose-dependent manner (Fig. 1B). The results implied that HCWEs might affected virus particles directly and result in the inhibition HSV-1 and HSV-2 infections.

### HCWEs inhibit viral entry

**Figure 2:**
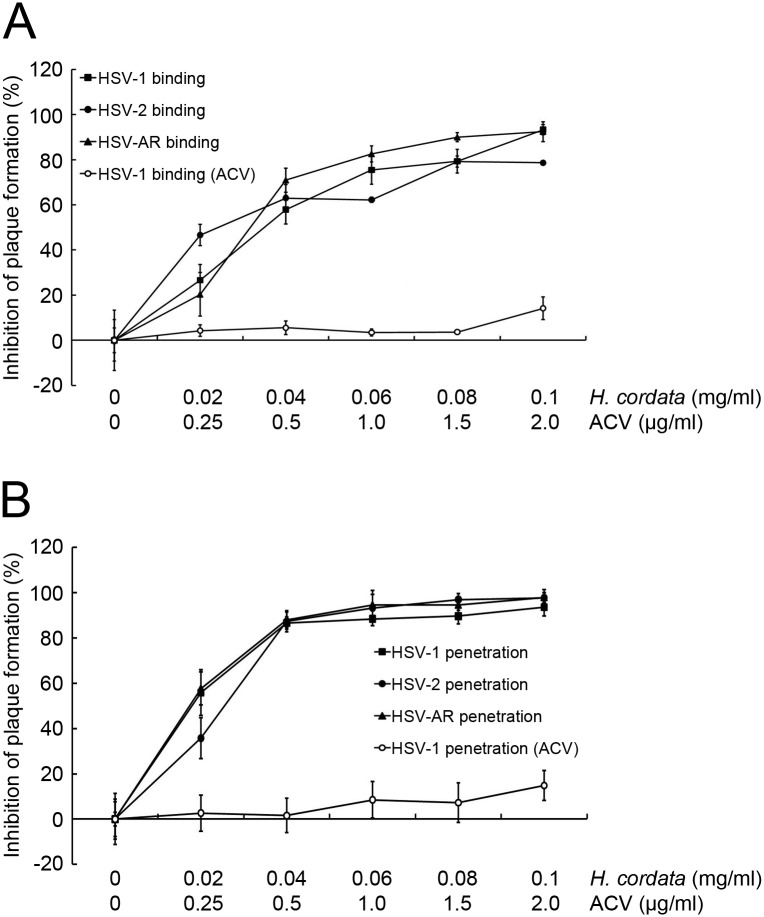
HCWEs inhibit HSV binding and penetration to host cells. **(A)** HCWEs inhibited virus binding to host cells. 1X10^6^ Vero cells were cooled to 4°C and exposed to 100 pfu HSV-1 (filled square), HSV-2 (filled circle), and HSV-AR (filled triangle) with indicated concentrations of HCWEs. Plaque assays were used to determine the inhibitory effect of HCWEs on the virus binding stage. **(B)** HCWEs inhibited virus penetration into host cells. 1X10^6^ Vero cells were infected with 100 pfu HSV-1 (filled square), HSV-2 (filled circle), and HSV-AR (filled triangle) for 2 hours at 4°C and then incubated with HCWEs at 37°C to allow viral penetration. Plaque assays were used to determine the inhibitory effect of HCWEs on the virus penetration stage.

**Figure 3:**
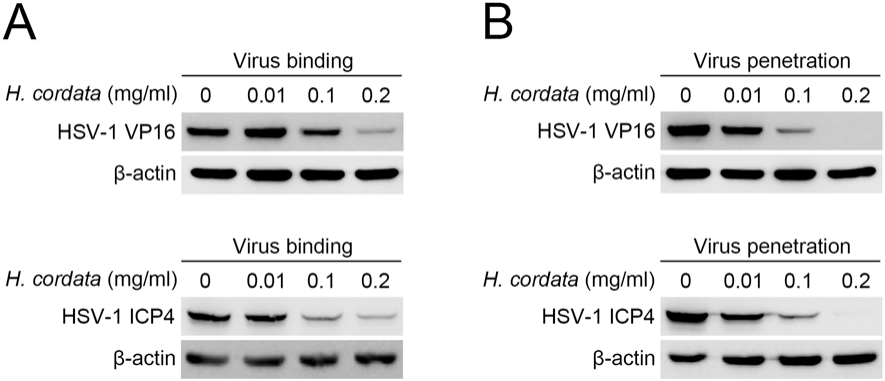
HCWEs inhibit HSV entry. HCWEs were added to Vero cells in virus binding **(A)** or penetration **(B)** stage as described in Fig. 2A and Fig. 2B. Nuclear extracts and total proteins were extracted from virus-infected cells and analyzed by Western blot assays with anti-VP16 and anti-ICP4 antibodies, respectively. β-actin was used as an internal control for protein loading.

HCWEs can interfere virus particles directly and inhibit HSV infection. To characterize the inhibitory mechanism of HCWEs and further identify which stage HCWEs act, the effects of HCWEs on viral binding and penetration were investigated separately. Vero cells were first cooled to 4°C and infected with viruses in presence of various concentrations of HCWEs for 2 hours. The unbound viruses and HCWEs were washed out with PBS, and the cell-bound viruses were then analyzed by plaque assays. As shown in Fig. 2A, HCWEs inhibited HSV-1, HSV-2, and HSV-AR binding ability in a dose-dependent manner. 0.1 mg/ml HCWEs inhibited 93.27%, 78.65%, and 92.44% of HSV-1, HSV-2, and HSV-AR plaque formation, respectively. ACV was served as a negative control because ACV is a guanosine analogue but does not block viral entry. The results indicated that the plaque formation was not obviously inhibited in the ACV-treated group. In addition to viral binding, we also examined whether HCWEs could influence viral penetration into host cells or not. Vero cells were cooled to 4°C and incubated with virus for 2 hours. The unbound viruses were washed out with PBS, and the cells were then incubated with HCWEs at 37°C for viral penetration. After 10 minutes of incubation, neutralization buffer was added to inactivate non-penetrated viruses and the virus-penetrated cells were analyzed by plaque assays. The percentages of inhibition on HSV-1, HSV-2, and HSV-AR penetration at a concentration of 0.1 mg/ml HCWEs were 93.62%, 97.76%, and 97.99%, respectively (Fig. 2B). To further precisely realize whether the reduction of plaque formation induced by HCWEs can be attributed to blockage of viral binding and penetration at least partly, we would determine the effect of HCWEs on viral entry directly. The translocation of viral tegument protein VP16 into the nucleus and the expression of viral immediate-early protein ICP4 have been used as the indicators to examine HSV entry [37]. HCWEs were added to the host cells at the viral binding or penetration stage as described in Fig. 2A and Fig. 2B, and the nuclear fraction and total proteins were extracted to examine the viral VP16 translocation and viral ICP4 expression by Western blot assays, respectively. As shown in Fig. 3A and Fig. 3B, viral VP16 translocation and viral ICP4 expression were inhibited by HCWEs in both the binding and the penetration stages.

### HCWEs block recombinant gD binding to host cells

**Figure 4:**
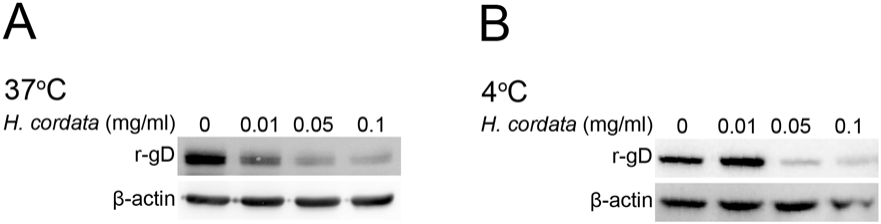
HCWEs block recombinant gD binding to host cells. 1X10^6^ Vero cells were incubated with recombinant gD (r-gD) proteins at 37°C **(A)** or 4°C **(B)** in the presence or absence of indicated concentrations of HCWEs. Cell lysates were analyzed by Western blot assays with anti-gD and anti-β-actin antibodies. β-actin was used as an internal control for protein loading.

We have demonstrated HCWEs could affect virus particles directly and inhibit virus infection while which viral envelope protein mediated the inhibitory activity was not identified. HSV glycoprotein D (gD) plays a critical role in viral binding onto host cells, cell-to-cell spreading, and viral entry into host cells [38–40]. To determine whether gD is a target of HCWEs, the recombinant gD proteins were expressed by baculovirus expression system and incubated with Vero cells at 37°C or 4°C for 1 hour to allow recombinant gD to bind onto Vero cells. The unbound recombinant proteins were washed out with PBS, and the bound recombinant proteins were detected by Western blot assays. As shown in Fig. 4A and Fig. 4B, HCWEs substantially inhibited the binding of recombinant gD onto Vero cells at concentrations of 0.05 mg/ml at both 37°C and 4°C. To rule the possibility that the decrease of recombinant gD binding is due to protein degradation caused by HCWEs, the recombinant gD proteins were incubated with different concentrations of HCWEs at 37°C for 3 hours, and analyzed by Western blot assay. Even in the highest concentration, HCWEs did not lead into gD degradation (S2 Fig.).

### HCWEs suppress HSV replication after viral entry

**Figure 5:**
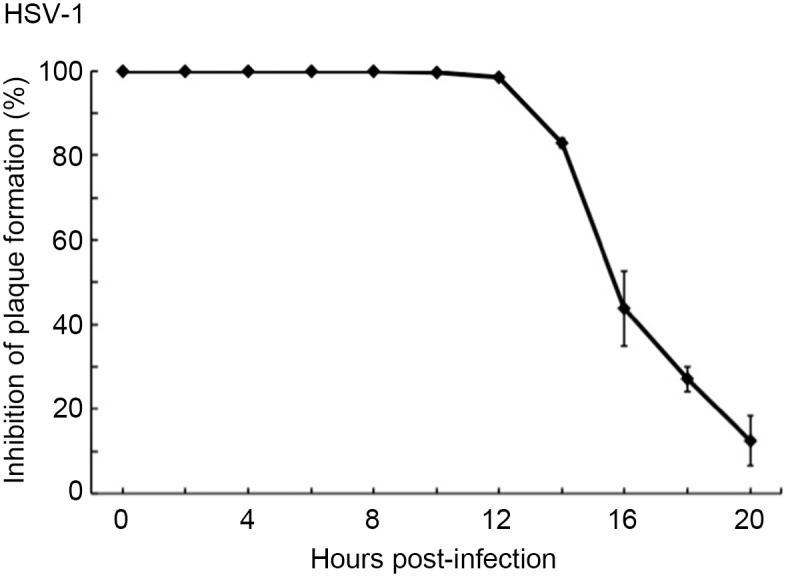
HCWEs inhibit viral plaque formation after HSV infection. 1X10^6^ Vero cells were infected with 1 m.o.i. HSV-1 at 37°C for 1 hour, and HCWEs (1 mg/ml) were added at different time points. Inhibitory effect of HCWEs on post virus infection was determined by plaque assays. h.p.i.: hours-post infection.

We demonstrated that HCWEs target HSV gD, interfere with viral binding and penetration, and finally block viral entry. To further explore whether is there other anti-HSV mechanism of HCWEs acting after viral entry, the time-of-addition experiments were performed. 1 mg/ml HCWEs were added at indicated hours post-infection (h.p.i.), and the cells were harvested at 24 h.p.i. The cell lysates were frozen and thawed three times and then titrated by plaque assay. Intriguingly, HSV was totally inhibited even if host cells were treated with 1 mg/ml HCWEs at 12 h.p.i. but not in the case of 0.01 mg/ml HCWEs (Fig. 5 and S3 Fig.). We next investigated whether HCWEs suppress virus replication through regulating viral late genes at this stage. The results indicated that the expression levels of viral late genes such as gB, gC, gD, gE, gH, and UL13 were downregulated in the presence of HCWEs (S3 Fig.).

### HCWEs suppress viral gene expression via inhibiting HSV-induced NF-κB activation

**Figure 6:**
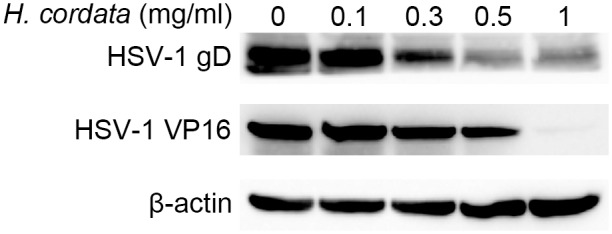
HCWEs shut down the protein synthesis of HSV-1. 1X10^6^ Vero cells were infected with HSV-1 at 1 m.o.i. in the presence or absence of indicated concentrations of HCWEs. Viral gD and VP16 were detected by Western blot assays with anti-gD and anti-VP16 antibodies. β-actin was used as an internal control for protein loading.

**Figure 7:**
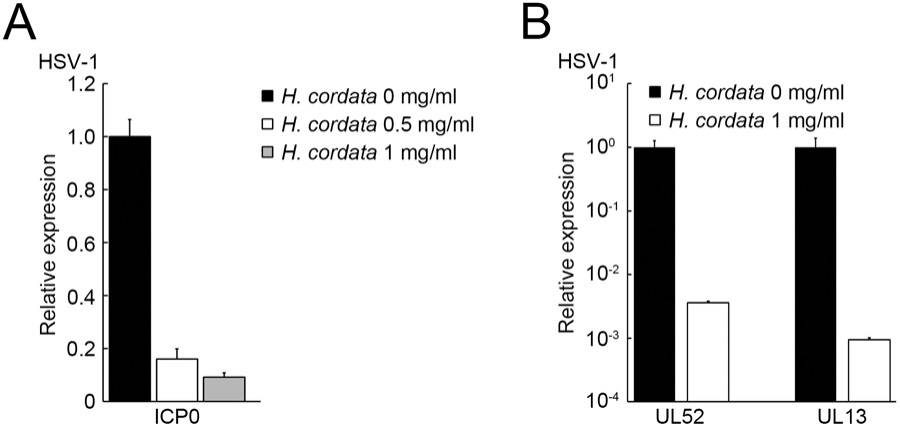
HCWEs suppress the gene expression of HSV-1. **(A)** 1X10^6^ Vero cells were infected with HSV-1 at 1 m.o.i. in the presence or absence of HCWEs. RNAs were extracted from the virus-infected cells at 2 h.p.i. and analyzed for viral ICP0 expression. **(B)** RNAs were extracted at 10 and 16 h.p.i. and analyzed for viral UL52 and UL13 gene expressions, respectively.

To address the stages in which HCWEs suppress HSV replication after viral entry, we first determined whether HCWEs influence the expression of viral proteins. Vero cells were first infected with HSV-1 followed by treatment of HCWEs for 24 hours, and the viral protein expression was detected by Western blot assay. As shown in Fig. 6, 1 mg/ml HCWEs obviously inhibited HSV-1 protein expression at 24 h.p.i.. HSV-1 IE genes are responsible for viral E and L gene expression and also essential for the viral lytic cycle. Thus, we next hypothesized that HCWEs could affect viral IE gene expression and further suppress viral proteins after viral entry. The host cells were infected with HSV-1 followed by treatment of HCWEs, and the RNA was extracted from HSV-infected cells at 2, 10, and 16 h.p.i. for analysis of the expression of the IE, E, and L genes, respectively, by real-time PCR (Fig. 7A and Fig. 7B). ICP0 expression, the viral IE gene, was inhibited by HCWEs in a dose-dependent manner (Fig. 7A). Besides ICP0, the expression of ICP4, ICP22, ICP27 and ICP47 was also determined and the results indicated HCWEs could regulate other viral IE genes expressions slightly (S4 Fig.). Furthermore, the expression levels of HSV-1 E gene UL52 and L gene UL13 were inhibited by HCWEs up to 300 and 1,000 folds, respectively (Fig. 7B). These data suggested that HCWEs might suppress the expression of viral genes through inhibiting viral IE gene ICP0 expression.

**Figure 8:**
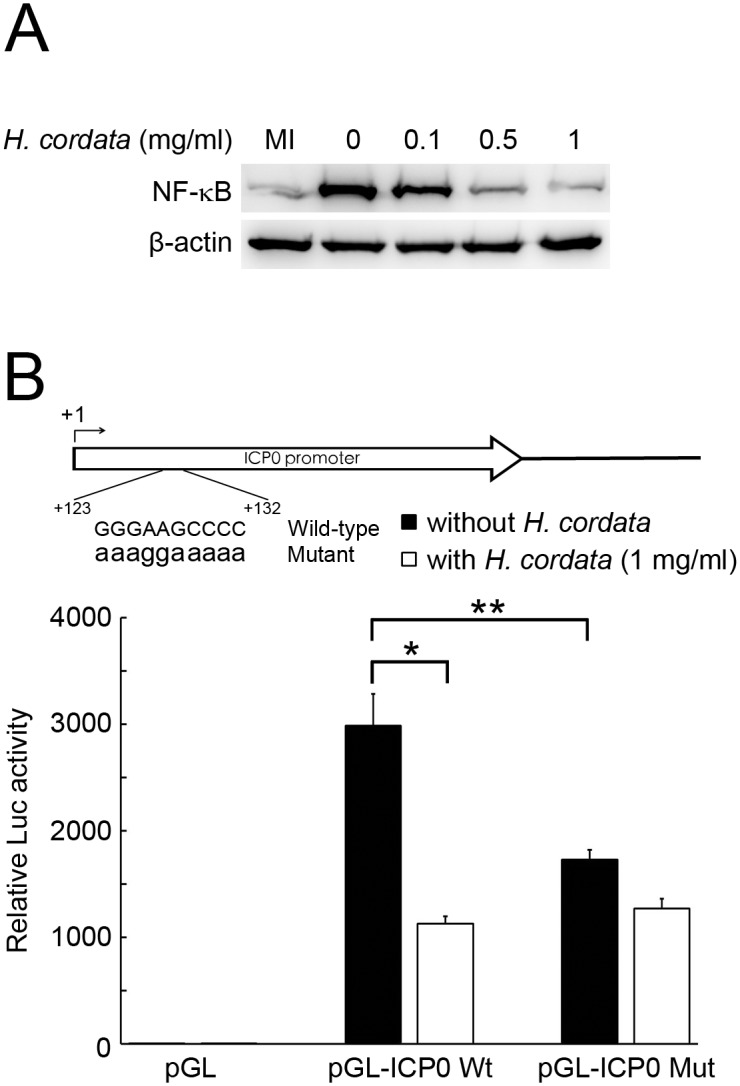
HCWEs eliminate HSV-1 induced NF-κB activation and suppress ICP0 transcription. **(A)** HCWEs inhibited HSV-induced NF-κB activation. 1X10^6^ cells were infected with HSV-1 at 1 m.o.i. in presence of HCWEs. The nuclear fraction was extracted at 3 h.p.i. and analyzed for NF-κB translocation. **(B)** HCWEs suppressed ICP0 transcription in NF-κB-dependent manner by promoter assays. 2X10^3^ cells were transfected wtih pGL reporter vectors harboring HSV-1 ICP0 promoter with wild-type (WT) or mutant (Mut) NF-κB binding core sequence and then infected with HSV-1 at 1 m.o.i. in the presence or absence of 1 mg/ml HCWEs. The luciferase activities were measured at 24 h.p.i.. * p value<0.05 presented comparison of pGL-ICP0 Wt in the presence or absence of HCWEs; ** p value<0.05 presented comparison of pGL-ICP0 Wt and pGL-ICP0 Mut without HCWEs.

During HSV infection, there are two waves of NF-κB activation. The first-wave of NF-κB activation could enhance ICP0 transcription via binding onto viral ICP0 promoter while the second-wave of NF-κB activation could prevent host cells from apoptosis [17]. It has been reported that HCWEs block the second-wave of NF-κB activation in HSV-2 infection [18, 29]. We hypothesized whether HCWEs could suppress the first-wave of NF-κB activation and further eliminate NF-κB binding onto HSV ICP0 promoter. To test this hypothesis, host cells were infected with HSV-1 in the presence of different concentrations of HCWEs, and the cell lysates were harvested for determination of NF-κB activity. The nuclear fractions were extracted and applied to NF-κB translocation analysis by Western blot assay. As shown in Fig. 8A, HCWEs inhibited NF-κB translocation in a dose-dependent manner. To further explore whether HCWEs could inhibit HSV-1 ICP0 promoter activity via inhibition of NF-κB activity, the HSV-1 ICP0 promoter was constructed into the pGL luciferase reporter vector, named as pGL-ICP0 Wt. After transfection, luciferase activity of pGL-ICP0 Wt transfected cells increased up to 3,000-fold compared with the vector control, pGL, in HSV-1 infection. However, the induction of luciferase activity was suppressed by HCWEs (Fig. 8B). Although it is well known the expression of ICP0 is regulated by NF-κB, the exact NF-κB binding site on ICP0 promoter is not identified yet. Hence, we first used TRANSFAC software to predict the potential NF-κB binding sites within HSV-1 ICP0 promoter. Only one NF-κB binding site was predicted within the HSV-1 ICP0 promoter. To determine whether the HCWEs-mediated ICP0 suppression was NF-κB-dependent, the predicted NF-κB binding site within pGL-ICP0 Wt was mutated by PCR-based mutagenesis and the resulting plasmid was named as pGL-ICP0 Mut. Fig. 8B showed that the luciferase activity of ICP0-Mut was significantly decreased compared with ICP0-Wt in HSV infection, while there was no obvious suppression between HCWEs treatment and not. The results implied that HSV-1 ICP0 promoter activity was at least partly regulated by NF-κB. To our best knowledge, besides viral ICP0 promoter, there were no potential NF-κB binding sites had been identified within other viral IE genes promoters and that might partially reason why other viral IE genes such as CP4, ICP22, ICP27 and ICP47 did not regulate under HCWEs treatment.

### HCWEs universally inhibit HSV replication in different cell lines

We demonstrated that HCWEs inhibit HSV infection in Vero cells in multiple stages, including viral binding, viral penetration, and viral genes expression. We then demonstrated these anti-viral activities are ubiquitous phenomena in different human cell lines. Two additional human epithelial carcinoma cells HEp-2 and A549 cells were tested for cytotoxicity and anti-HSV activity by the plaque reduction assay. HCWEs showed no cytotoxicity on HEp-2 and A549 cells at 100 mg/ml (CC_50_ > 100 mg/ml). Importantly, the plaque reduction assays showed that HCWEs inhibited HSV-1 and HSV-2 replication in both HEp-2 and A549 cells with an EC_50_ similar to that of Vero cells (S1 Table).

### Major compounds of HCWEs possess anti-HSV activity

**Figure 9:**
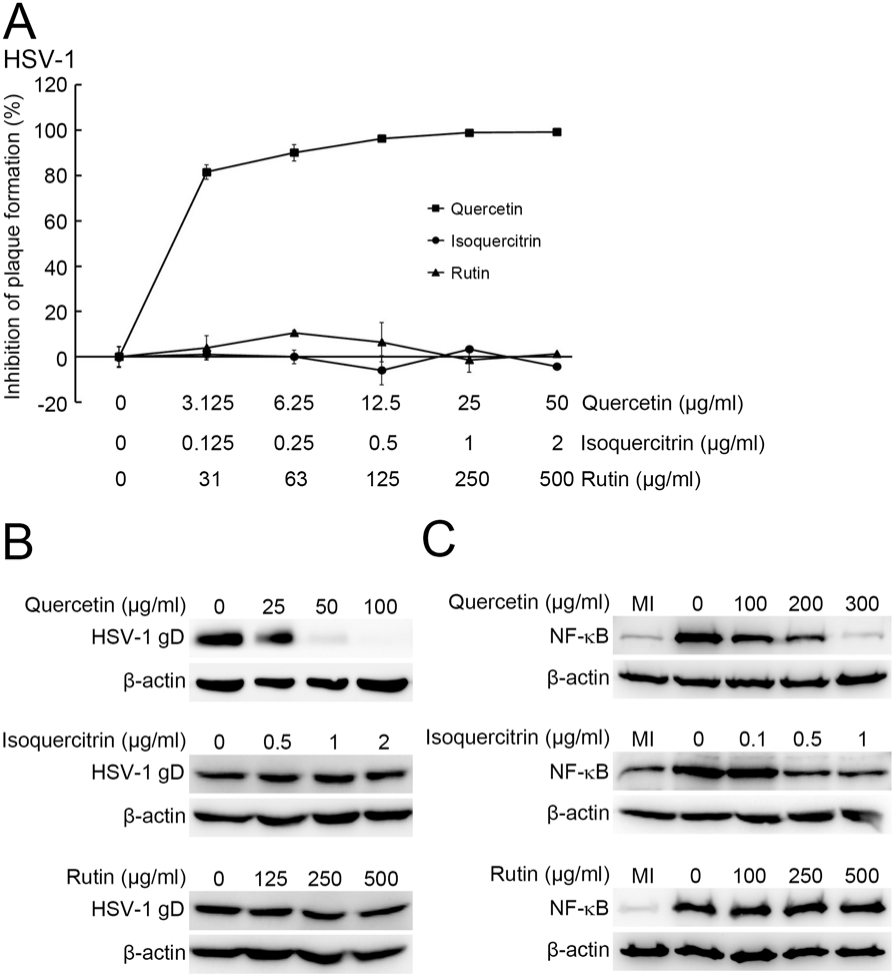
Analysis of anti-HSV mechanism of rutin, quercetin, and isoquercitrin. **(A)** Quercetin inhibited HSV-1 infection before viral entry. 1X10^6^ Vero cells were infected with 100 pfu HSV-1 pretreated with rutin (filled triangle), quercetin (filled square), and isoquercitrin (filled circle), and plaque assays were used to determine the inhibitory effects. **(B)** Quercetin inhibited the binding of HSV-1 onto host cells. 1X10^6^ Vero cells were infected with HSV at 1 m.o.i. and incubated with quercetin, isoquercitrin or rutin at 4°C for 2 hours. The viral gD protein was detected by Western blot assay. β-actin was used as an internal control for protein loading. **(C)** The inhibitory effect of quercetin, isoquercitrin and rutin on HSV-induced NF-κB activation. 1X10^6^ cells were infected with HSV-1 and incubated with indicated concentrations of quercetin, isoquercitrin or rutin. The nuclear fractions were extracted at 3 h.p.i. and analyzed for NF-κB translocation. β-actin was used as an internal control for protein loading.

The major components of HCWEs have been identified as chlorogenic acid, hyperin, rutin, quercetin, quercitrin, and isoquercitrin [41]. Quercetin, quercitrin, and isoquercitrin showed anti-HSV activity but which stage of HSV replication affected was not elucidated clearly [27]. To determine which stage these compounds act on, the pure components were analyzed by plaque reduction assays. Among these 6 compounds, rutin, isoquercitrin, and quercetin demonstrated anti-HSV-1 activities, with EC_50_ of 187.58 μg/ml, 0.42 μg/ml, and 52.9 μg/ml, respectively (S2 Table). We further studied the anti-HSV mechanism of each active compound. The virus pretreatment assays and virus binding assays showed that quercetin, but not rutin or isoquercitrin, targeted virus particles directly and inhibited viral entry (Fig. 9A and Fig. 9B). Furthermore, we also identified which compound contributed to inhibition of NF-κB activity by Western blot assay. As shown in Fig. 9C, both isoquercitrin and quercetin inhibited NF-κB activation in HSV infection.

**Figure 10:**
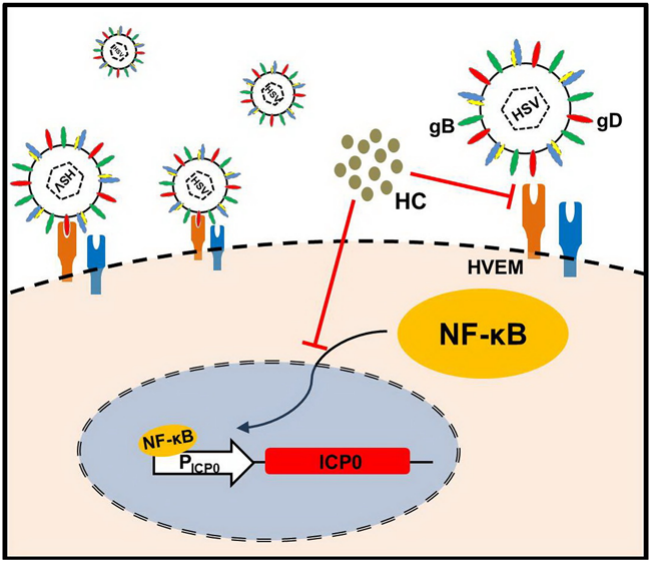
Anti-HSV-1 mechanism of HCWEs. HCWEs inhibit HSV infection at multiple stages. First, HCWEs affect viral gD binding onto host cells and further influence HSV infection. Second, HCWEs suppress the transcriptional activity of ICP0 promoter through eliminating HSV-induced NF-κB activation.

In summary, we discovered a new anti-HSV mechanism of HCWEs by which HCWEs target to virus particles and block viral binding and penetration in HSV infection. Additionally, HCWEs also inhibited ICP0 promoter expression through suppressing HSV-induced NF-κB activation, thereby suppress the expression of viral genes and block the synthesis of viral proteins (Fig. 10).

## Discussion

In this study, besides HSV-1 and HSV-2, our data demonstrated that HCWEs even inhibit HSV-AR replication. It shed light on the development of new anti-HSV drugs for overcoming the surge of drug resistance in HSV. HCWEs affected multiple stages of HSV infection, such as viral entry and viral gene expression, and thus eventually suppress HSV replication. Furthermore, quercetin possesses anti-HSV activities, particularly on inhibition of viral binding, viral penetration, and host NF-κB activation, and isoquercitrin suppresses NF-κB activity. Rutin, another major component of *H. cordata*, has been shown to have anti-HSV activity, with an EC_50_ of 187.58 μg/ml. However, the anti-HSV mechanism of rutin is still unclear and needs to further investigate.

Pretreatment of HSV with HCWEs blocked viral plaque formation, but no obvious inhibitory effect was observed in HCWEs-pretreated host cells. The experiments of the ICP4 expression and the VP16 translocation further confirmed that HCWEs inhibited viral entry in both the binding and the penetration stages. HSV gD protein can bind to four coreceptors up to date, including herpesvirus entry mediator (HVEM), nectin-1, nectin-2, and 3-*O*-sulfated heparin sulfate, which is necessary for viral post-binding entry into host cells [38–40]. UV-inactivated wild-type HSV virions, but not UV-inactivated virions lacking gD, and soluble gD could block HSV infection implied that viral gD is an ideal target for developing anti-HSV drugs [42–45]. HCWEs inhibited the binding of recombinant gD protein onto host cells. The fact indicated that HCWEs may act on HSV gD protein directly and inhibit viral entry at the stages of viral binding and penetration. Certainly, we could not exclude the possibility that other viral glycoproteins such as viral gB, gC, gH and gL might involve in the inhibition of viral entry.

After viral entry, HCWEs inhibited viral IE genes, E genes, and L genes expressions. Furthermore, HCWEs suppressed HSV-induced NF-κB activation and attenuated NF-κB mediated ICP0 transcription. ICP0, one of the HSV IE genes, could bind to several cellular proteins as well as viral promoters resulting in increased expression of viral E and L genes [46–48]. Thus, ICP0 is an important gene for the expression of viral E gene and L gene, and it can further regulate HSV replication. VP16, a tegument protein of HSV, can bind to the host cell factor (HCF) and form the VP16-HCF complex. The VP16-HCF complex then binds to the cellular protein, OCT-1, and stimulates the transcription of ICP0. In addition to the transactivating activity of VP16, other cellular cis-acting elements are also important in regulating the expression of ICP0, one of them being NF-κB. NF-κB is a host transcriptional regulator and can be activated in almost all types of cells by many types of stimulations, such as inflammation, bacterial and viral infections [49]. During HSV infection, there are two waves of NF-κB activation. The first-wave was triggered through the binding of viral gD to the cellular receptor, and the activated NF-κB was recruited onto viral ICP0 promoter to enhance ICP0 expression [17]. In the second-wave activation, NF-κB was activated after 6 hours post-infection and could prevent host cells from apoptosis [50]. The compounds with inhibition of NF-κB activation have been reported to exhibit anti-HSV activity. Resveratrol and Lornixicam could downregulate HSV infection-induced NF-κB and further impair viral IE genes expressions and prevent recurrent herpetic stromal keratitis, respectively [51]. It was demonstrated that HCWEs could block HSV infection through inhibiting the second-wave of NF-κB activation and our results revealed that HCWEs could also inhibit the first-wave of NF-κB activation. However, in the time-of-addition assay we found that 0.01 mg/ml HCWEs could not inhibit viral plaque formation. In contrast to post-treatment, pre-treatment with same dose could suppress HSV replication. These evidences suggested that the inhibitory activity of HCWEs against HSV might mainly come from the HCWEs pre-treatment. Taken together, these evidences suggested that HCWEs not only inhibit HSV infection in early stages such as viral binding and penetration but also suppress virus replication via governing viral late genes after viral entry. However, whether other inhibitory mechanisms such as suppression of viral DNA synthesis and block of viral package also involved in suppressing virus replication at this stage were needed to further investigate.

As we know, the therapeutic efficacy of herb extracts is quite inconsistent due to the differences in herb species, production place, and extraction procedure. It was why we would like to identify what is the key component of HCWEs contributing to antiviral activity. The quality of pure compound is much easier to control compared with crude extracts. We found that rutin, quercetin, and isoquercitrin exhibit anti-HSV activities. Previous reports have indicated that these three compounds have anti-HSV activities, though the molecular mechanisms of each active compound were still unclear [27, 52–54]. To the best of our knowledge, ours is the first report to present the anti-HSV mechanisms of quercetin and isoquercitrin. In the present study, Western blot and virus pretreatment assays revealed that only quercetin could block the binding of HSV to host cells. The NF-κB translocation assays indicated that quercetin and isoquercitrin blocked HSV replication by inhibiting NF-κB activation in virus infection. However, the anti-HSV mechanisms of rutin are still unclear. Moreover, the SI value of isoquercitrin to HSV was greater than 512.8 in this study, indicating a potential target for drug development.

Quercetin and isoquercitrin, both flavonol compounds having similar structures, are widely distributed in nature. Quercetin has been shown to have an inhibitory effect on lipopolysaccharide-induced nitric oxide synthase gene expression and to inhibit NF-κB activation in human synovial cells, primary cultured rat proximal tubule cells, and rat aortic smooth muscle cells [55–57]. Isoquercitrin, a glucose-bound derivative of quercetin, has also been shown to have anti-oxidant and anti-inflammation properties [58]. Recently, these flavonol compounds have been reported to have anti-viral activity against viruses such as influenza, poliovirus, adenovirus, respiratory syncytial virus, SARS coronavirus, and HIV [59–61]. HSV infection can induce oxygen stress, and the viral ICP27 protein can induce apoptotic cell death by increasing cellular reactive oxygen species (ROS) [62, 63]. In this report, quercetin and isoquercitrin are shown to inhibit HSV-induced NF-κB activation. Further studies are needed to clarify the correlation between the anti-oxidant properties and the anti-HSV activities of quercetin and isoquercitrin. Quercetin is a small molecule, and our study showed that it could inhibit HSV binding to the cell surface. We hypothesize that this small molecular compound may compete for the gD binding site with a cellular receptor such as heparin sulfate or nectin-1. However, more work will be needed to test this hypothesis.

## Supporting Information

S1 Fig The effect of HCWEs-pretreated Vero cells on HSV-1 infection. 1X10^6^ Vero cells were seeded onto 6-well plates, treated with HCWEs and then infected with 100 pfu/well HSV-1. The inhibitory activities were analyzed by plaque assay.
(TIF)

S2 Fig The effect of HCWEs on gD protein degradation *in vitro*. Recombinant gD proteins were incubated with different concentrations of HCWEs at 37°C for 3 hours, and analyzed by Western blot assay.
(TIF)

S3 Fig The effects of HCWEs on virus replication and viral late genes expressions after HSV infection. **(A)** 1X10^6^ Vero cells were infected with 1 m.o.i. HSV-1 at 37°C for 1 hour, and 1 mg/ml (filled diamond) or 0.01 mg/ml (unfilled diamond) HCWEs were added at different time points. Inhibitory effect of HCWEs on post virus infection was determined by plaque assays. h.p.i.: hours-post infection. **(B)** HCWEs suppress viral late genes expressions after HSV infection. 1X10^6^ Vero cells were infected with HSV-1 at 1 m.o.i. in the presence or absence of HCWEs. RNAs were extracted from the virus-infected cells at 16 h.p.i. and analyzed for viral late genes expressions.
(TIF)

S4 Fig The effect of HCWEs on viral IE genes. 1X10^6^ Vero cells were infected with HSV-1 at 1 m.o.i. in the presence of HCWEs. RNAs were extracted from the virus-infected cells at 2 h.p.i. and analyzed for viral ICP0, ICP4, ICP22, ICP27 and ICP47 expressions.
(TIF)

S1 Table Anti-HSV activities of HCWEs. (DOC)

S2 Table Anti-HSV activities of major compounds contained of *Houttuynia cordata*. (DOC)
