# Supplementary figures and images for: Houttuynia cordata Targets the Beginning Stage of Herpes Simplex Virus Infection
Source: PLoS One. 2015 Feb 2;10(2):e0115475. doi: 10.1371/journal.pone.0115475 (PMC4314066; doi:10.1371/journal.pone.0115475)

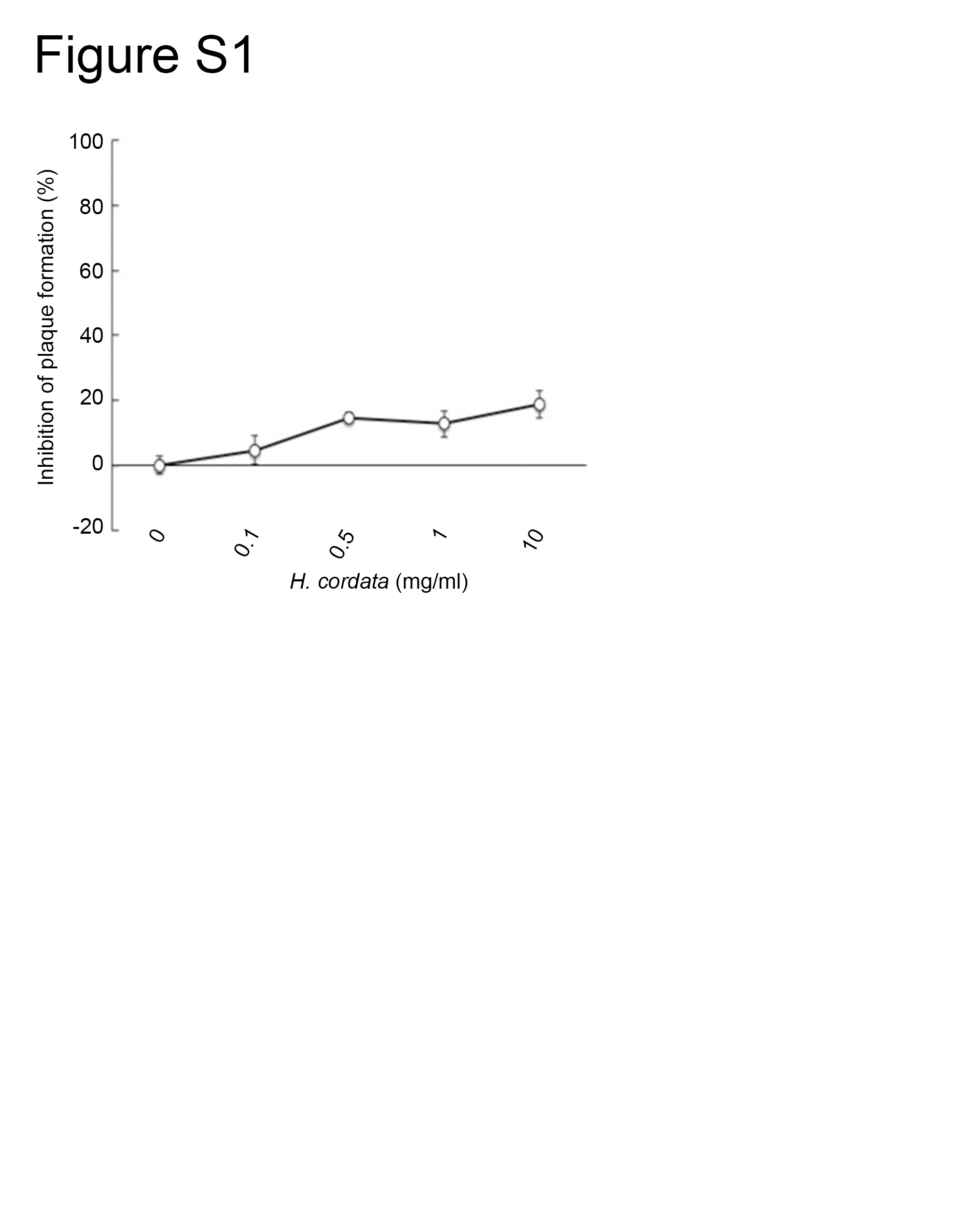

Supplement: S1 Fig — 1X106 Vero cells were seeded onto 6-well plates, treated with HCWEs and then infected with 100 pfu/well HSV-1. The inhibitory activities were analyzed by plaque assay. (TIF) [file pone.0115475.s001.tif]

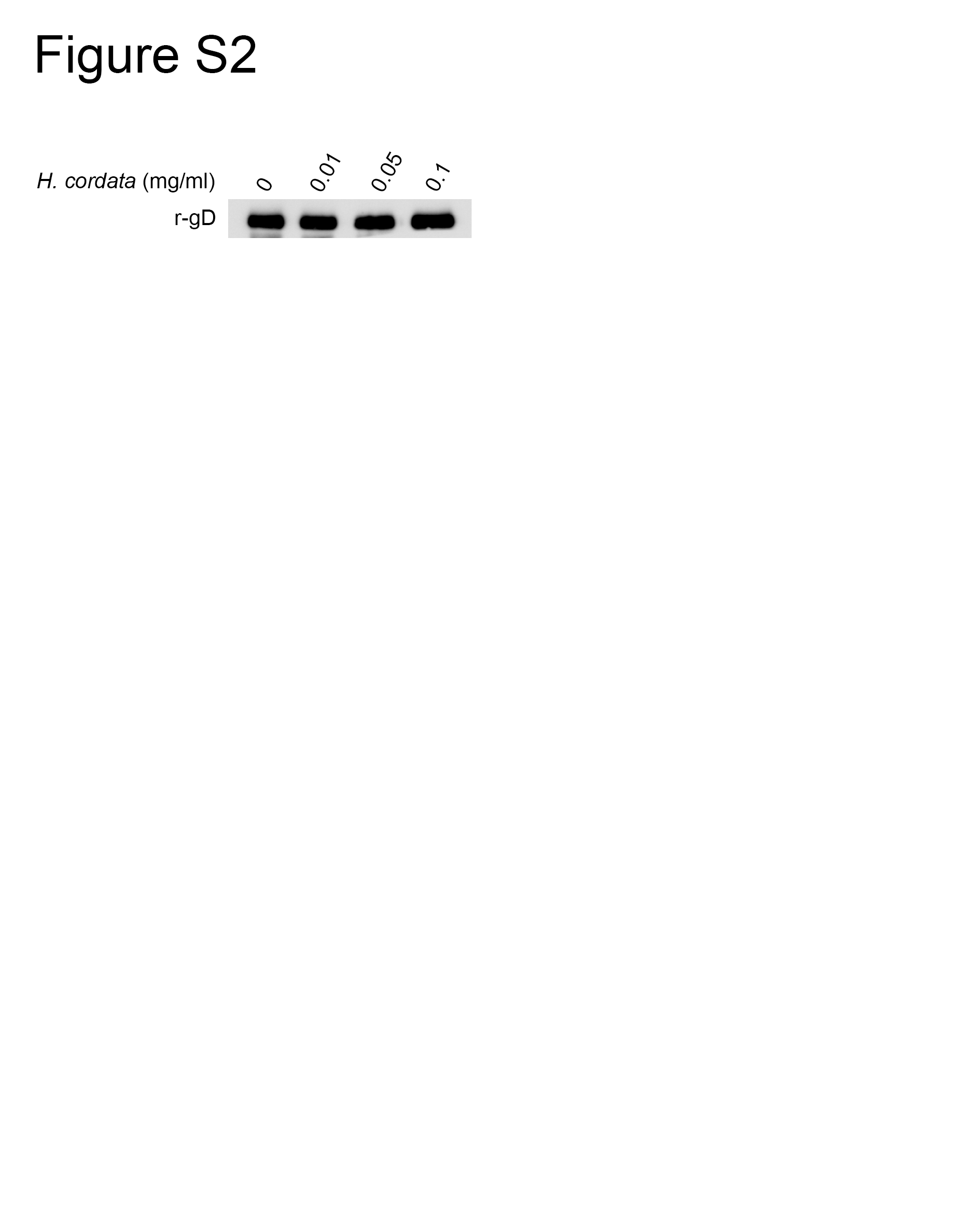

Supplement: S2 Fig — Recombinant gD proteins were incubated with different concentrations of HCWEs at 37°C for 3 hours, and analyzed by Western blot assay. (TIF) [file pone.0115475.s002.tif]

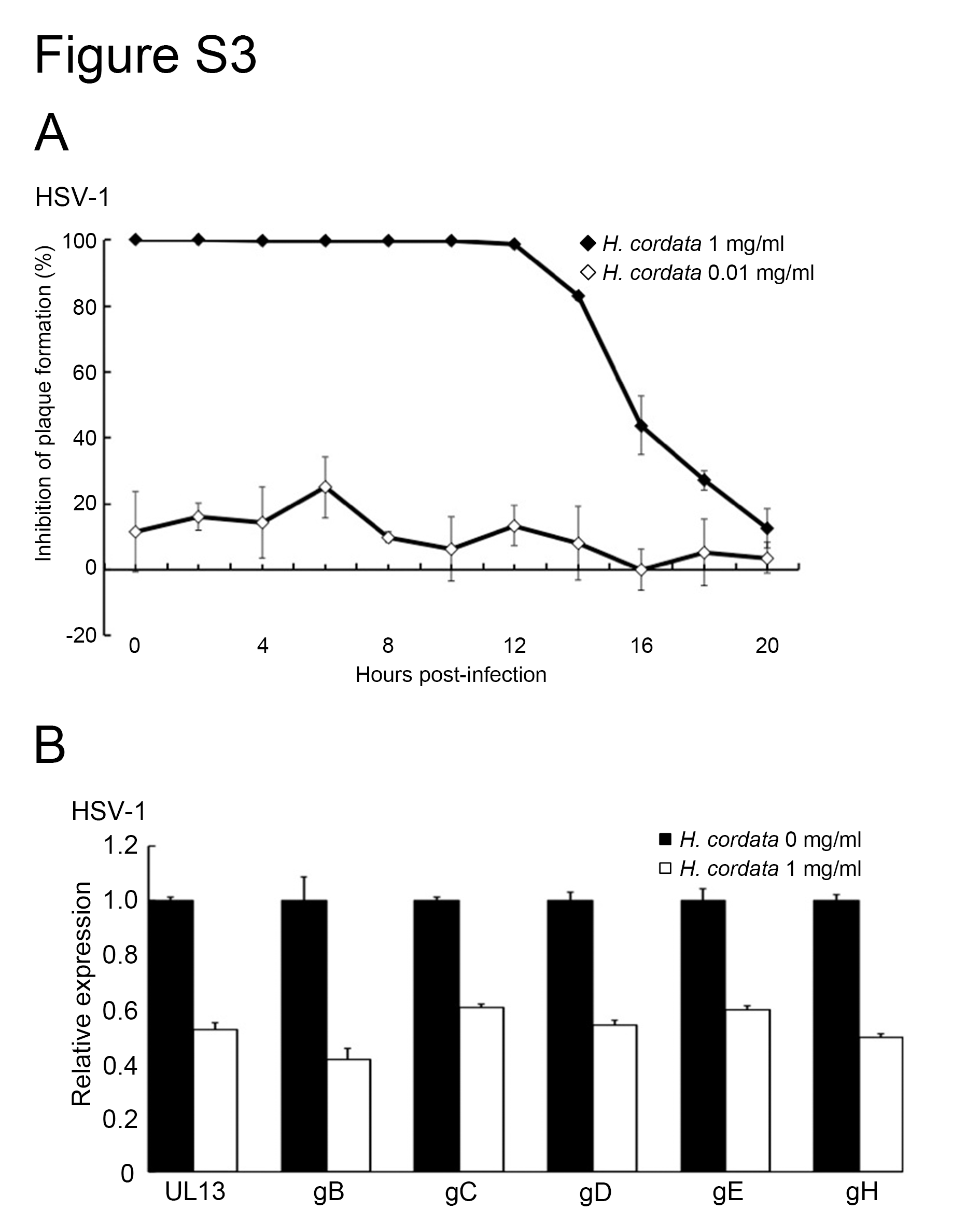

Supplement: S3 Fig — (A) 1X106 Vero cells were infected with 1 m.o.i. HSV-1 at 37°C for 1 hour, and 1 mg/ml (filled diamond) or 0.01 mg/ml (unfilled diamond) HCWEs were added at different time points. Inhibitory effect of HCWEs on post virus infection was determined by plaque assays. h.p.i.: hours-post infection. (B) HCWEs suppress viral late genes expressions after HSV infection. 1X106 Vero cells were infected with HSV-1 at 1 m.o.i. in the presence or absence of HCWEs. RNAs were extracted from the virus-infected cells at 16 h.p.i. and analyzed for viral late genes expressions. (TIF) [file pone.0115475.s003.tif]

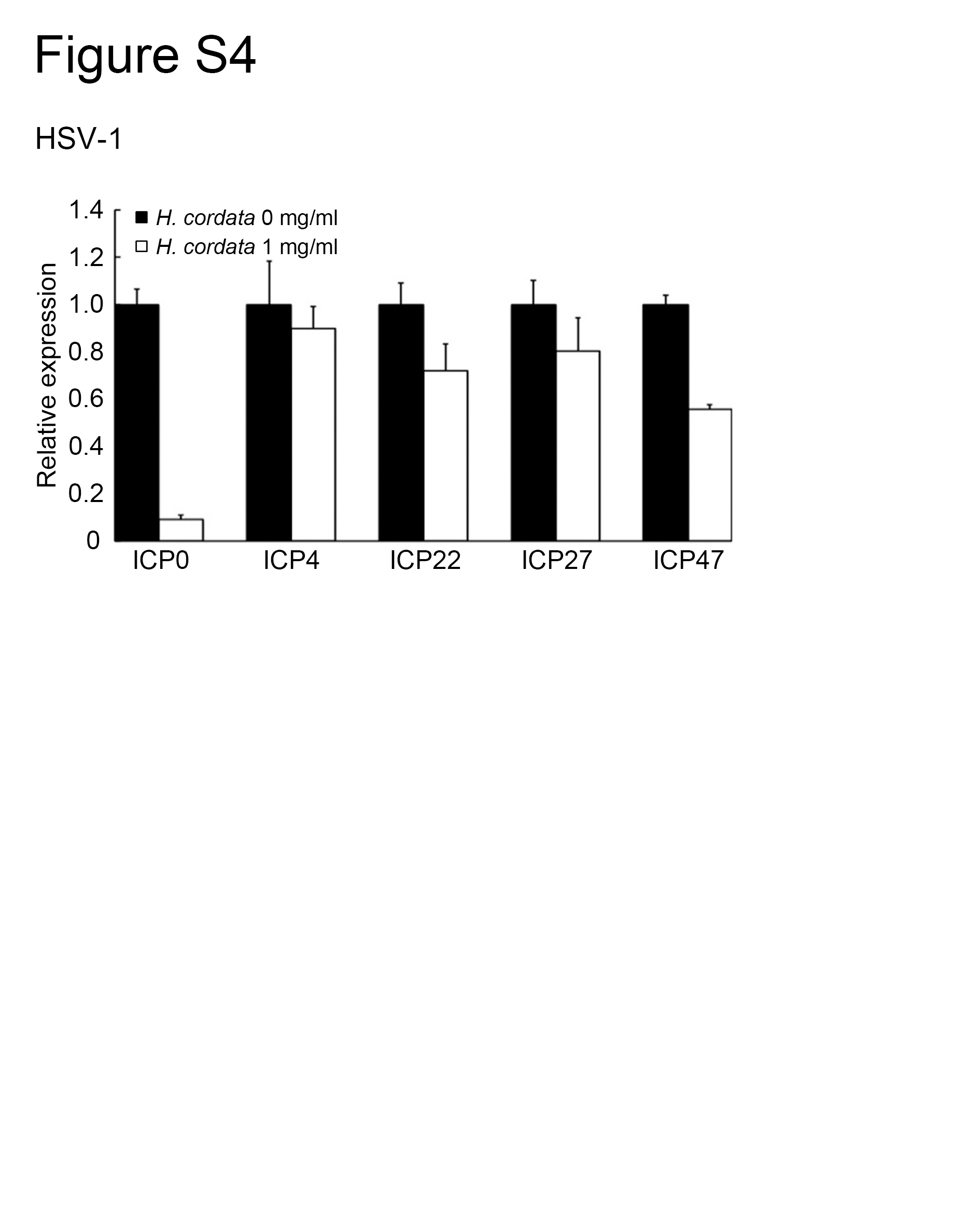

Supplement: S4 Fig — 1X106 Vero cells were infected with HSV-1 at 1 m.o.i. in the presence of HCWEs. RNAs were extracted from the virus-infected cells at 2 h.p.i. and analyzed for viral ICP0, ICP4, ICP22, ICP27 and ICP47 expressions. (TIF) [file pone.0115475.s004.tif]
