# Supplementary material for: Houttuynia cordata Targets the Beginning Stage of Herpes Simplex Virus Infection
Source: PLoS One. 2015 Feb 2;10(2):e0115475. doi: 10.1371/journal.pone.0115475 (PMC4314066; doi:10.1371/journal.pone.0115475)
Supplement: S1 Table — (DOC) [file pone.0115475.s005.doc]

| Cell | Drug | CC_50_^a^ |  | HSV-1 | |  | HSV-AR | |  | HSV-2 | |
| --- | --- | --- | --- | --- | --- | --- | --- | --- | --- | --- | --- |
|  |  |  |  | EC_50_^b^ | SI^c^ |  | EC_50_^b^ | SI^c^ |  | EC_50_^b^ | SI^c^ |
| Vero | Houttuynia cordata (mg/ml) | >100 |  | 0.692 | >144.51 |  | 1.11 | >90.09 |  | 0.3 | >333.33 |
| HEp-2 | Houttuynia cordata (mg/ml) | >100 |  | 0.41 | >243.9 |  | 0.191 | >523.56 |  | 0.147 | >680.27 |
| A549 | Houttuynia cordata (mg/ml) | >100 |  | 0.31 | >322.58 |  | 0.260 | >384.62 |  | 0.151 | >662.25 |
| Vero | Acyclovir (μg/ml) | >450 |  | 0.74 | >608.11 |  | 14.02 | >32.1 |  | 0.82 | >548.78 |

**Table S1 Anti-HSV activities of HCWEs**

Values in this table represent the mean of three independent experiments.

^a^ CC_50_ was the concentration that showed 50% of cytotoxic effect in assayed cells.

^b^ EC_50_ was the concentration that inhibited 50% of HSV replication in assayed cells.

^c^ The selective index (SI) was calculated as CC_50_/EC_50_.
