# Supplementary material for: Houttuynia cordata Targets the Beginning Stage of Herpes Simplex Virus Infection
Source: PLoS One. 2015 Feb 2;10(2):e0115475. doi: 10.1371/journal.pone.0115475 (PMC4314066; doi:10.1371/journal.pone.0115475)
Supplement: S2 Table — (DOC) [file pone.0115475.s006.doc]

***Table S2 Anti-HSV activities of major compounds contained of Houttuynia cordata***

| Compound | CC_50_^a^ (μg/ml) |  | HSV-1 | |  | HSV-2 | |
| --- | --- | --- | --- | --- | --- | --- | --- |
|  |  |  | EC_50_^b^ (μg/ml) | SI^c^ |  | EC50 (μg/ml) | SI |
| Quercitrin | 142.16 |  | >142.16 | -^d^ |  | >142.16 | - |
| Quercetin | 485.69 |  | 52.9 | 9.18 |  | 70.01 | 6.94 |
| Chlorogenic acid | 1243.07 |  | >1243.07 | - |  | >1243.07 | - |
| Hyperin | >500 |  | >500 | - |  | >500 | - |
| Rutin | 702.45 |  | 187.58 | 3.745 |  | 169.42 | 4.146 |
| Isoquercitrin | >200 |  | 0.42 | >476.2 |  | 0.39 | >512.8 |

Values in this table represent the mean of three independent experiments.

^a^ CC_50_ was the concentration that showed 50% of cytotoxic effect in Vero cells.

^b^ EC_50_ was the concentration that inhibited 50% of HSV replication in Vero cells.

^c^ The selective index (SI) was calculated as CC_50_/EC_50_.

^d^ - presented SI could not be calculated.
